# Automatic trajectory planning for stereotactic radiofrequency ablation in non-discrete search space

**DOI:** 10.1007/s11548-025-03386-1

**Published:** 2025-05-11

**Authors:** Adela Lukes, Reto Bale, Wolfgang Freysinger

**Affiliations:** 1 Interventional Oncology-Stereotaxy and Robotics (SIP), Radiology Department, Medical University Innsbruck, Innsbruck, Austria; 2 4D Visualization Laboratory, ENT Clinic, Medical University Innsbruck, Innsbruck, Austria

**Keywords:** Radiofrequency ablation, Trajectory planning, Genetic algorithm, Continuous search space

## Abstract

**Purpose:**

Radiofrequency ablation is a well established minimally invasive procedure to treat tumors in solid organs. During the procedure applicators are inserted into the tumor and cells around their tips are destroyed by heat-induced denaturation. Manual trajectory planning requires a trained interventionalist, and its complexity and planning time rise significantly with an increasing number of trajectories.

**Methods:**

We propose a trajectory planning method using a genetic algorithm to accelerate the planning process by automatically generating multiple safe plans. Our method uses a non-discrete search space to find the best entry and target points and does not need any prior calculation of such candidate’s points sets. The method offers multiple plans, allowing the interventionalists to choose the most appropriate one. We tested on an open-source and in-house dataset, comparing with related work and retrospectively with the in-house clinical planning.

**Results:**

Our method, tested on 154 liver tumors across all segments using a 10 mm ablation radius, achieves a mean coverage of over 99% of the tumors including a 5 mm safety margin. The method provides safe trajectories for all solutions and is on average 4$\times$ faster than related approaches.

**Conclusion:**

To the best of our knowledge, we are the first to propose a fast and accurate planning technique using multiple applicators with 10 mm ablation radius. Our algorithm can deliver solutions optimizing more than ten trajectories, approaching the clinical practice at our institution, where large tumors are treated with multiple overlapping ablation zones rather than resection.

## Introduction

Cancer affects millions of individuals globally and is the second leading cause of death in the USA, liver cancer ranking as the fourth most common malignancy [2]. Radiofrequency ablation (RFA) offers a minimally invasive method to treat tumors in liver or other solid organs. In this procedure, an ablation applicator is precisely inserted into the tumor in the organ and the cells around its uninsulated tip are destroyed.

In most clinics, RFA approach is supported as a treatment of small liver tumors. Stereotactic radiofrequency ablation (SRFA), developed at our clinic for the treatment of larger tumors with several overlapping ablation zones, is utilized infrequently [3]. Our center is one of the few where SRFA is deeply exploited, pushing the limits of the ablation of large tumors with up to 30 applicators. Nonetheless, concurrently planning several trajectories while guaranteeing their safety is challenging, rendering SRFA not readily scalable to other clinics without extensive training [4].

SRFA is conducted under general anesthesia, with all pertinent procedures executed during maximal expiration. A contrast-enhanced computed tomography (CT) scan with 1 mm slice thickness is obtained and this dataset is sent to the stereotactic navigation system. Path planning is carried out manually. Subsequently, coaxial needles are inserted according to the plan. Accurate needle placement is verified by an unenhanced CT scan and image fusion. Thereafter, sequential ablation using a total of three ablation probes is performed. A final contrast enhanced CT scan and image fusion is used to evaluate whether the tumor including a sufficient safety margin is covered by the ablation zone.

We propose an algorithm that automatically creates plans for liver tumor ablation without any manual input, such as defining or computing a fixed set of possible target points. Moreover, parameters, e.g., ablation radius, are adjustable, allowing the users to adjust the algorithm to their available hardware. Our genetic algorithm, yields more than one suitable plan, allowing the clinicians to pick the solution that fits their needs the most.

## Related work

Algorithms for trajectory planning for ablation techniques have been only sparsely proposed due to the problem complexity and reliance on accurate segmentations. This section examines significant planning methods, encompassing not only radiofrequency techniques but also microwave and cryoablation (MWA, CA), as the primary distinction is in the ablation radius of the applicator, while the planning processes remain analogous. Refer to  [5] for a comprehensive review of keyhole surgery advancements up to 2019.

The early approaches were solving the problem with a single trajectory suitable for small tumors only. In practice, a safety margin of 5–10 mm has to be applied to reduce the risk of tumor recurrence. Thus, a single RFA probe with a 1 cm ablation zone radius can ablate a tumor with a maximum diameter of 1 cm. MWA creates a more extensive ablation zone (up to 2 cm), enabling it to treat a tumor with a maximum diameter of about 2 cm. Various software aiming to ease the planning has been proposed since the early 2000 s [6–8]. Baegert et al. proposed a downhill-simplex method for single trajectory planning utilizing occlusion from the critical structures to find suitable insertion points on the skin [9]. Seitel et al. presented a system for planning optimal trajectories to a predefined target based on 3D reconstructions with GPU implementation for efficiency [10]. Schumann et al. simulated the heat distribution around the probe tip incorporating the cooling effect from the nearby blood vessels [11].

Planning multiple trajectories simultaneously depends on multiple interdependent target points, meaning altering one target point influences the placement of others. Ant-colony optimization was presented by Giorgi et al. [12] for CA planning for prostate cancer for multiple probes and evaluated on a phantom. Chen et al. proposed a clustering method for a better layout of the target points for RFA treatment with multiple probes and evaluated their approach on 18 scans and two clinical cases [13]. Luo et al. proposed a planning method using attraction and repulsion fields [14], and a Gaussian-based strategy is proposed for CA optimizing up to 4 trajectories [15].

The pullback technique minimizes the number of punctures by creating multiple ablation zones with one needle. The applicator, after ablation at its furthest target, is pulled back and another ablation zone is created. This allows the interventionalists to create a cylindrical shape of the ablation zone and minimize the number of applicators used. Liang et al. utilized a cover-set based method for the automatic planning with multiple probes and pullback [16]. Li et al. proposed a heuristic approach that utilizes a simplified approach for trajectory planning in 2D projections rather than in 3D [17], initializing the optimization search along the long axis of the tumor.

Genetic algorithms (GAs) were first applied to trajectory planning in 2021, when Yu et al. [18] proposed a method based on selected candidate target points and precomputed sets of safe trajectories for each. Approximating the ablation zone as an ellipsoid, the authors use a set of 12 common ablation zone sizes, the largest of them being $40 \times 40 \times 45$ mm, from which the algorithm can choose when optimizing. The optimal combination of the precomputed paths is found using GA. Pullback technique and GA is used in the work of Li et al. [19]. They initially determine the ablation number by grouping the tumor points and subsequently constrain the permissible puncture area accordingly. Nevertheless, scant information was offered regarding the context and application of the GA.

**Fig. 1:**
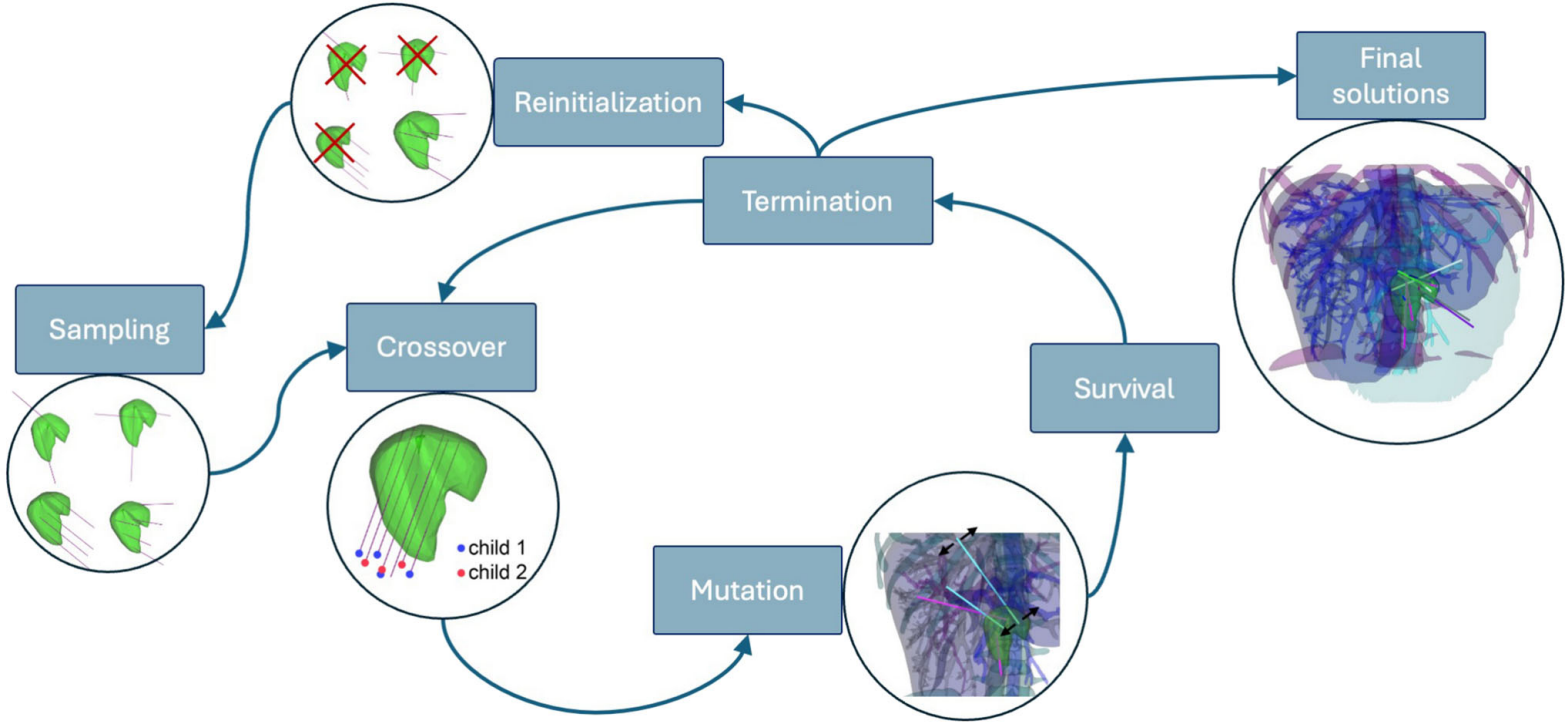
Flowchart depicting our genetic algorithm for SRFA. The sampling operator generates the first population of potential solutions. The tumor is illustrated in green. The left circle illustrates the initial population of solutions. The crossover and mutation operators later modify this population. The survival operator selects the most advantageous individuals from both the parent and offspring populations. The termination criterion monitors the convergence of the optimization solutions, as depicted in the rightmost circle

## Methods

Our algorithm, illustrated in Fig. 1, is using a non-dominated sorting genetic algorithm II (NSGA-II) [20] which avoids deleting the individuals on the Pareto front. It selects the individuals frontwise by dominance and crowding distance, supporting diversity within the population. Mating selection with binary tournament for crossover and survival operator use this sorting process. We keep these operators for sorting and survival, while we tailor the sampling, crossover, and mutation to our problem. Moreover, to enhance the method’s robustness against local minima, we introduce a reinitialization to the GA process, where new individuals are generated using our sampling operator. Algorithm 1 delineates the comprehensive framework of the process, whereby the inputs comprise the environment, including the population size (popSize), and supplementary parameters (params) such as the radius of the ablation zone. The environment defines the entry space (skin) and target space (tumor and safety margin), liver, and critical structures.

**Algorithm 1:**
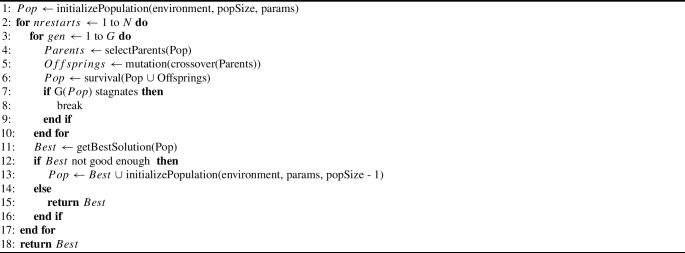
Genetic algorithm

In general, a constrained optimization problem is defined as

$$ \underset{x \in S \subseteq \mathbb {R}^n}{\text {minimize}} \hspace{1mm} F(x), \hspace{5mm} \text {subject to} \hspace{5mm} G(x) = 0. $$

We model the individuals of our population as a long array *x*, in which entry and target point of each trajectory are sequentially saved. The cost function *F* consists of 6 functions *f* and the constraint function *G* of 2 functions *g*. We normalize all functions to [0, 1] and transform the functions that should be maximized by taking $1-$value. The definitions are as follows:

- $f_0$: mean trajectory length (minimize),
- $f_1$: mean trajectory length in tumor (maximize),
- $f_2$: mean trajectory length in liver (minimize but keep longer than 5 mm),
- $f_3$: mean angle of the trajectories to the normal of the liver and skin surface (minimize),
- $f_4$: coverage of the tumor and margin (maximize),
- $f_5$: number of trajectories (minimize),
- $g_0$: binary value, 1 if the trajectories in the solution intersect critical structures or other trajectories and 0 otherwise,
- $g_1$: coverage of the tumor and margin, which is 0 if the $f_4$ is smaller than the $\varepsilon$-threshold.

**Fig. 2:**
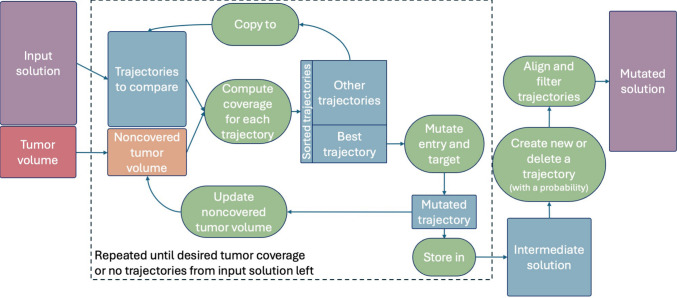
Data flow diagram of mutation. In each iteration of the main loop (dashed rectangle), trajectories from the input solution are sorted with respect to their tumor coverage. The highest-ranking trajectory is selected, mutated, and stored in an intermediate solution. The residual noncovered treatment volume is used in the next iteration together with the remaining trajectories. The loop continues until either the desired coverage is reached or no trajectories remain from the input solution. If non-covered tumor volume exists a completely new trajectory is created. Similarly, with a certain probability is the worst-performing trajectory deleted from the intermediate solution. The final step ensures that the trajectories conform to physical constraints of the problem environment

We model the ablation zone as a cylinder, a commonly used simplification for the pullback technique. Our sampling operator creates trajectories along the longest tumor axis, that are as parallel as possible, as illustrated in Fig. 1, focusing on the pullback and the habit of inserting the needle as parallel as possible, as colliding probes can lead to a short circuit during the ablation.

The simulated binary crossing operator is ill-suited for our case, as it generates children element-wise by combining the values of the parents. Such approach unlikely creates suitable children. An illustrative scenario are parent trajectories going below and above a rib. A combination of their entry points with binary crossover would presumably produce an unacceptable trajectory colliding with the rib. Therefore, our crossover method selects the trajectories from the parents in their entirety without altering their coordinates. The trajectories are chosen to maintain a safe distance from one another while providing the best coverage of the treatment volume.

**Fig. 3:**
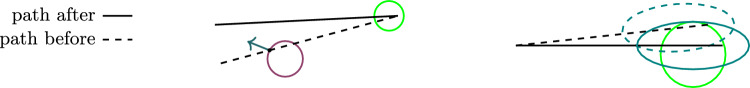
Mutation of a colliding (left) and non-colliding (right) trajectory. The green circles represent the treatment volume. The dashed lines represent trajectories prior to mutation, whereas the solid lines denote those subsequent to mutation. The magenta circle depicts a crucial structure. The algorithm calculates the normal vector of the critical structural mesh surface at the intersection point (shown by the blue arrow), and the trajectory proceeds in this direction. On the right, the non-colliding trajectory is altered to include a greater extent of the tumor. Ellipses depict the ablation zones

Our mutation operator consists of multiple steps illustrated in Fig. 2. We modify the initial solution, by iteratively selecting and mutating trajectories from the input to maximize the tumor coverage with minimal number of trajectories, while minimizing collisions with critical structures. Figure 3 illustrates individual trajectory mutation called “mutate entry and target” in Fig. 2. If collision occurs, the trajectory moves with a repulsive force in the direction of the normal vector at the intersection of the obstacle mesh. Otherwise, mutation varies the entry and target on a small scale and compares if such alterations result in better coverage. After the main loop, the algorithm adds a new trajectory if tumor coverage is insufficient, and, with certain probability, removes the worst-performing trajectory. The final stage of mutation aligns the entrance locations accurately on the patient’s skin, while the target points remain inside the treatment volume. It additionally excludes too short trajectories (< 5 mm in the liver) and excessively steep angles (angle $< 20^\circ$ to the tangent of the skin and hepatic capsule), while shortening very long trajectories (length > 150 mm).

### Reinitialization of the process

No fixed set of target or entry points is computed or manually defined prior to the optimization, and the search space remains non-discretized. This reflects clinical practice, where interventionalists adjust targets and entry points dynamically on CT scans based on feasibility and tumor coverage. Our method mirrors this approach, enabling planning consistent with real-world procedures. The extensive search space constitutes the primary obstacle, as excessive research may hinder convergence. To mitigate, we restrict excessive exploration by implementing crossover and mutation operators that permit only minimal modifications.

We avoid numerous local minima in the search space by restarting the process. Monitoring the sum of the constraint function *G*, the algorithm reinitializes in case of stagnation throughout multiple iterations, which is identified by the standard deviation of the minimum value. The reinitialization process keeps the best candidate and the rest of the population is created with the sampling operator, introducing more diversity into the population.

## Data & data preprocessing

We evaluate on an open-source 3D-IRCADb-01 (IRCAD) [1] and in-house dataset. IRCAD consists of 20 segmented cases of liver, tumors, skin, and liver vessels. We exclude cases number 5, 7, 11, 12, 14, and 20 due to the absence of liver tumors. Case 12 has an exophytic lesion. From case 6, we exclude the largest tumor spreading over multiple liver segments. No other restrictions were used, e.g., maximal tumor diameter or position, in order to provide challenging cases and investigate the robustness of the algorithm.

For the in-house clinical dataset, we selected 20 recent patient cases from over 800 patients who were successfully treated with SRFA at our University Hospital [21]. For each subject, we combined the automatic segmentation [22] with manual correction and segmentation of the liver structures. Afterward, a clinician examined the segmentations of liver, tumor, and liver vasculature rating them on a 1–5 scale (5 best). For evaluation, we picked cases with a tumor segmentation score of at least 3, which makes 38 tumors with volume up to 63.3 cm$^3$. The commonly identified drawback was the coarseness of the automatic masks, as [22] operates on lower-resolution CT scans. However, this error is reduced through smoothing during mesh creation.

We represent all abdominal structures in both datasets as meshes and utilize the VTK toolkit to generate oriented bounding box trees [23]. We filter the skin based on its proximity to the tumor, retaining the section closer to the tumor centroid than the applicator length of 150 mm. We categorize abdominal structures into four groups: tumor, liver, skin, and essential structures. Critical structures include various organs, ribs, and the vascular system. The tumor mesh is enlarged by 10 mm to incorporate the safety margin and is intersected with the liver mesh. The vasculature mesh is excluded from the expanded volume to create a realistic treatment volume.

## Evaluation

The evaluation of our algorithm was done on a Ubuntu OS with 8 Intel Core i7-9700K processors, RTX3070 32 GB GPU. However, our Python implementation with pymoo library [24] uses only CPU, which allows the algorithm to run on any system.

**Fig. 4:**
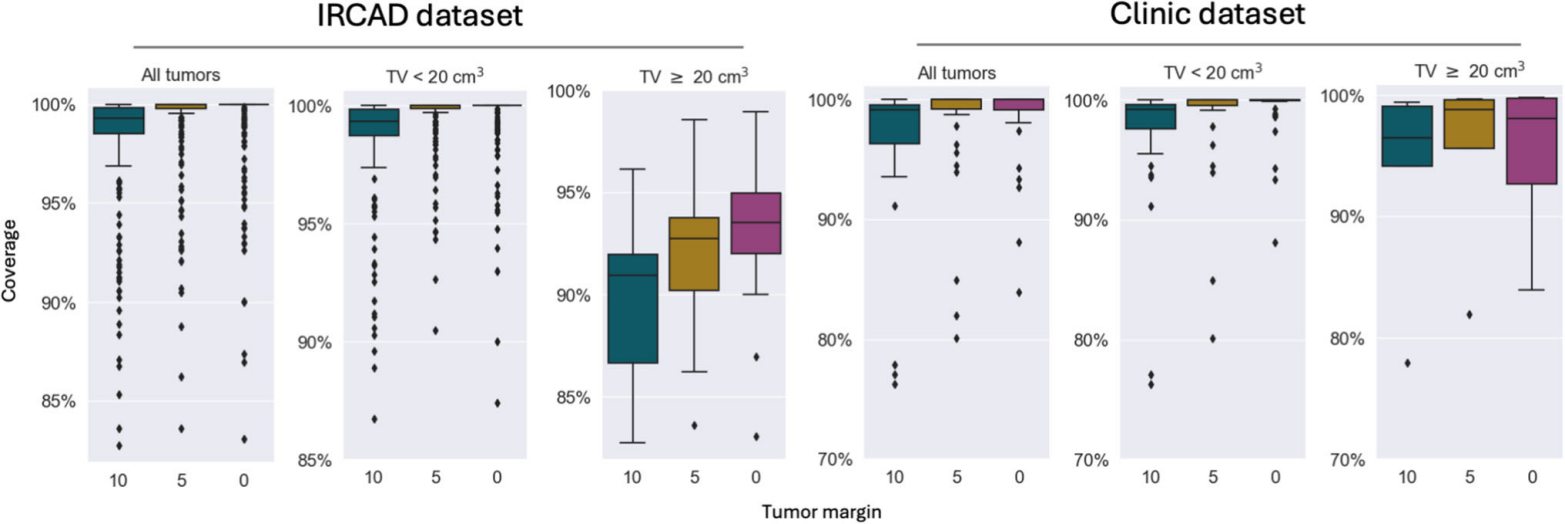
Boxplot comparisons of tumor coverage among three groups for the IRCAD and clinical datasets. For each dataset, the left boxplot illustrates the overall distribution of tumor coverage, whilst the middle and right boxplots represent coverage for small and big tumors, respectively. Be aware that the ranges of the y-axis vary amongst the graphs

The assessment was twofold. Initially, we assessed the methodology using the IRCAD dataset. Subsequently, we tested on actual tumor cases from our clinic and compared our solutions with those provided by the radiologists. The ablation radius was 10 mm, equal to a single RFA applicator. A maximum of 10 restarts was permitted, and the probability of eliminating the least favorable needle was set to 0.35. The algorithm successfully generated non-colliding solutions for all 154 examined tumors, and the resultant coverage is illustrated in Fig. 4.

**Table 1:** Comparison of the clinical and automatic solutions

	NT $\downarrow$	C10 $\uparrow$	C5 $\uparrow$	C0 $\uparrow$	SN $\sphericalangle$ $\downarrow$	L (mm) $\downarrow$
**Case 1**, TV: 6.44						
Clinical solution	5	0.790	0.948	0.993	55.1$^\circ$	67.20
Automatic solution 1	7	0.887	0.980	1.0	17.5$^\circ$	68.85
Automatic solution 2	6	0.862	0.969	1.0	20.05$^\circ$	71.12
Automatic solution 3	5	0.817	0.968	1.0	13.78$^\circ$	64.72
**Case 2**, TV: 9.31						
Clinical solution	5	0.624	0.755	0.913	33.41$^\circ$	124.28
Automatic solution	6	0.763	0.801	0.881	19.89$^\circ$	137.30
**Case 3**, TV: 1.38						
Clinical solution	6	0.898	0.987	1.0	23.76$^\circ$	57.08
Automatic solution	4	0.962	1.0	1.0	23.91$^\circ$	61.9

In the first column, TV means tumor volume in cm$^3$. Number of trajectories (NT), coverage of the tumor and its 10, 5, and 0 mm margins (C10, C5, C0) fill the first three columns, where 1.0 infers $100\%$ coverage. In the fourth column, we calculate the mean of the angles to skin normal at insertion points (SN). Lastly, we compute the mean length of the trajectories (L). The arrow by each column name indicates whether a lower or higher value is preferred

### Evaluation on IRCAD

For all 116 tumors in the IRCAD dataset, the computed trimmed mean of tumor coverage, with a $10\%$ outlier threshold, was $99.0\%, 99.8\%$, and $99.8\%$ for tumors with 10 mm, 5 mm, and 0 mm margins, respectively, as illustrated in Fig. 4. We compared references [18] and [16], as both employ the pullback technique and assess bigger tumors, with the results presented in Table 1. In [16], clinicians evaluated 20 solid tumors from 9 patients at IRCAD, each having a diameter of less than 6 cm and no proximity to vital structures. In [18], the authors selected 32 tumors from 9 IRCAD cases without disclosing the selection criteria. The authors of [18] assessed tumors measuring up to 44 cm³ utilizing an ablation zone of $40 \times 40 \times 55$ mm. In [16], the authors employ an ablation zone radius of up to 55 mm, with the maximum tumor volume in their sample being 28 cm$^3$.

Our methodology yielded non-collisional solutions for all 116 tumors with a volume of up to 83.5 cm$^3$, with a mean coverage of 99.2%. Table 2 presents results on subsets of our evaluation dataset, focusing on tumors with volumes smaller than the maximum volume reported in comparable studies to ensure a more accurate comparison. The authors did not specify the tumors used for evaluation, making direct replication of their results infeasible. Our algorithm employed 18 trajectories for a tumor with a volume of 27.6 cm$^3$, utilizing a 10 mm ablation radius. In comparison, [18] used four trajectories for a 44 cm$^3$ tumor, and [16] used seven for a 28 cm$^3$ tumor.

**Fig. 5:**
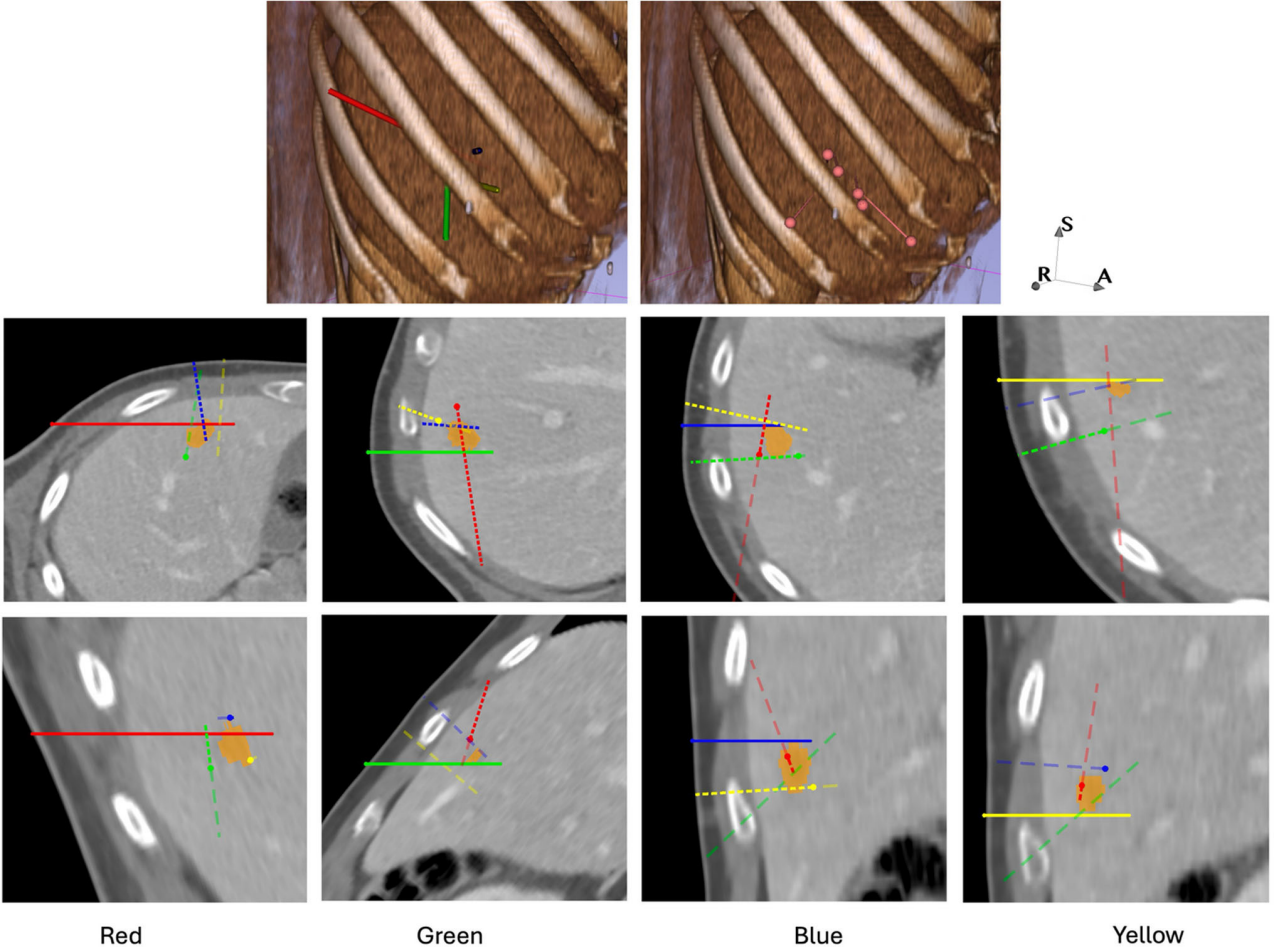
Solution for Case 3. The 3D renderings in the initial row depict the automatic solution on the left and the clinical solution on the right. An orientation marker is provided to elucidate the perspective of the 3D display. The ribs are distinctly apparent in light brown, with the lines representing the trajectories; multicolored indicates automatic, while pink signifies clinical solution. In the second and third rows, we display two perspectives for each trajectory of the automated solution on the CT image. The tumor is delineated in orange. Observe that in each column, only one trajectory exists in the plane, while the others are projections. The dashed lines illustrate the anticipated trajectories: the segment above the plane is indicated with short, entirely opaque dashes, while the component below the plane is represented with longer, semi-transparent dashes. Intersections of trajectories with the plane are denoted as a circle

**Fig. 6:**
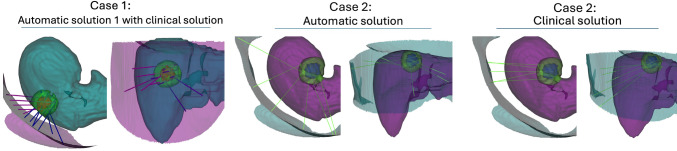
Solutions for Cases 1 and 2. For each solution, we present a cranial-to-caudal view (left) and a lateral right-to-left view (right). We generate 3D representations of the paths, skin, liver, tumor, and its 10 mm safety margin. The left graphic displays the automatic solution 1 for Case 1 (blue lines) alongside the clinical solution (pink lines). The middle and right images depict Case 2, showcasing the automatic and clinical solutions, respectively

### Evaluation on in-house dataset

Our methodology yielded non-colliding solutions for all 38 tumors. Figure 4 presents the assessment of coverage attained on the internal dataset of 38 tumors. The mean coverage of the tumors with 5 mm margin was 0.98$\pm 0.04$ and median 1.0. The mean number of ablations per applicator was 1.4 for the clinical solutions and 1.3 for the automatic algorithm. The average computation time was 8.5 minutes, and the median was 3.98 minutes. We provide a quantitative assessment of the relationships among the variables in the Appendix. Table 1 presents a detailed examination of three cases from our clinical dataset. The computation durations for Cases 1, 2, and 3 were 7.42, 16.98, and 2.2 minutes, respectively. Case 3 is depicted in Fig. 5, while the 3D renderings of Cases 1 and 2 are presented in Fig. 6.

**Table 2:** Quantitative comparison between related methods and our method

		Test dataset		Results		
	AZR (mm)	N	TV ($\hbox {cm}^3$)	MC (TMC) $\uparrow$	*p* value	T (min) $\downarrow$
[18]	up to 40	32	44.47	0.992 (1.0)	0.01	–
Ours	10	113	57.84	0.995 (0.999)		1.5
[16]	up to 55	20	28.38	1.0 (1.0)	0.016	8
Ours	10	112	27.6	0.996 (0.999)		1.3
Ours	10	116	83.48	0.992 (0.998)		2.1

In the columns we introduce the approach, the ablation zone radius (AZR), and the number of samples in test dataset (N) with the maximal tumor volume in the dataset (TV). The discrepancy between the maximal volumes in the fourth column may arise from different approaches of volume computation. In the last columns, we show the mean (MC) and the 10$\%$ trimmed mean (TMC) coverage of tumor and its 5 mm safety margin, where 1.0 infers $100\%$ coverage, and the *p* value from the Mann–Whitney U test for coverage. Lastly, we provide the average computation time in minutes (T). The “–" means that the value was not provided. The arrow by each header indicates whether a lower or higher value is preferred

The tumor in Case 3, Fig. 5, was located in liver segment 5 and measured 2 cm in diameter. The automated solution surpassed the clinical solution in this instance. The physicians employed 6 trajectories with 10 ablation positions, while our automated method attained complete tumor coverage and a 5 mm safety margin utilizing 4 trajectories with 6 positions. An expert radiologist evaluated the automated plans on the CT slices depicted in Fig. 5 and validated that it is an appropriate plan. A 3D rendering movie is included in the additional online material.

## Discussion

The proposed method offers feasible choices for SRFA with a minimal ablation zone and pullback. It offers noncolliding solutions for all 154 tumors and accommodates up to 18 trajectories simultaneously, specifically intended for RFA single applicators, unlike prior studies that need clustered applicators to get such extensive ablation zones. The identical algorithm can be readily modified for MWA applicators by altering the ablation radius to between 10 and 20 mm, contingent upon the special type of MWA.

Figure 4 illustrates the comparison of coverage attained on the IRCAD and clinical datasets. Our technique attained over 99% coverage on small IRCAD tumors; however, its efficacy on large tumors was inferior. The disparity in attained coverage between small and large tumors in the clinical sample was negligible. This presumably occurs because not all tumors in IRCAD are appropriate for SRFA, which our approach is designed for, while the in-house data solely comprises such tumors.

Although employing single RFA probes that produce ablation zone volumes five times smaller, our approach remains highly competitive with related work. It attains comparable coverage outcomes and generates secure plans while traversing an environment with several trajectories simultaneously. The p values in Table 2 indicate that our method marginally exceeded that of [18], whereas the method in [16] demonstrates a marginal improvement compared to ours. In contrast, the authors of the former evaluated only accessible cancers, while our technique was tested on tumors without limitations regarding location or proximity to important structures. Despite the necessity for our approach to concurrently optimize many trajectories, it typically delivers a solution in 2 minutes, which is four times quicker than [16]. The authors of [18], do not disclose calculation time; yet, their methodology seems computationally demanding. They precompute all potential paths and their feasibility for each target point within the treatment volume, which appears unnecessary as not all paths are utilized in the genetic algorithm. However, their subsequent optimization appears computationally efficient and likely executes quickly.

When tested on the in-house dataset, our algorithm yields competitive solutions to the clinical solutions, e.g., in Case 1. Observing Fig. 6, we see that the algorithm aligns with the clinical practice and tends to insert the probes in parallel. The left image in the figure shows that the algorithmic solution is comparable to the clinical. Moreover, as demonstrated for Case 3, the algorithm is able to propose clinically valid, safe solution with 100$\%$ coverage using less trajectories than the clinicians. However, for Case 2, our algorithm does not provide a superior solution, because the tumor is located in the subcapsular space near the lung and in proximity to large vessels (visualized in Appendix), which makes the planning particularly challenging.

**Fig. 7:**
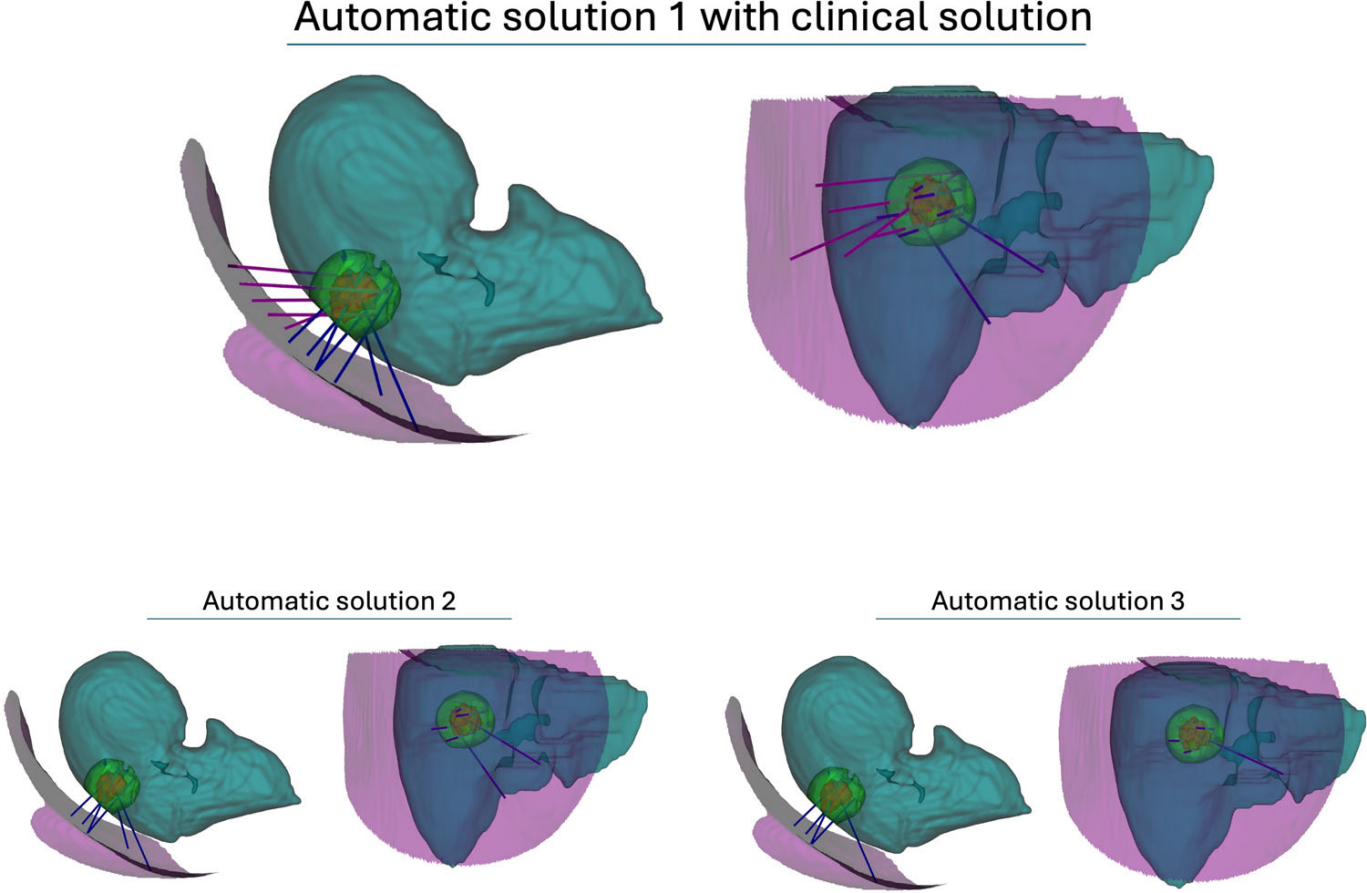
We show the 3D model and the trajectories of the solution for Case 1 presented in Results section. For each solution, we show cranial-to-caudal view on the left and lateral right-to-left view on the right. In the visualization, the skin is depicted (pink), liver in teal, tumor in red and its 10 mm safety margin in green. The first row shows in two views the automatic solution 1 (blue) and the clinical solution (pink). In the second row, we show the automatic solutions 2 (left) and 3 (right) in two views

**Fig. 8:**
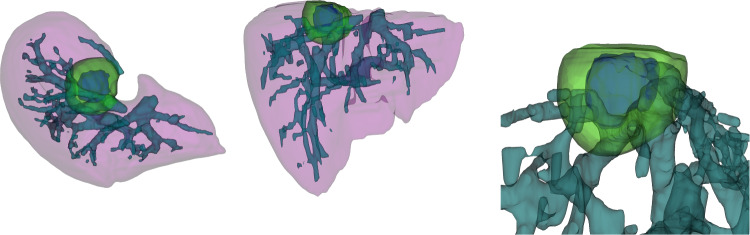
3D model of tumor, liver, and vessels for clinical Case 2. We show cranial-to-caudal view on the left and lateral right to left view on the right. In the visualization the liver is depicted in pink, vessel tree in teal, tumor and its 10 mm safety margin in blue and green, respectively. The right image shows a zoomed in 3D rendering of the tumor and the surrounding vessels in lateral view

**Fig. 9:**
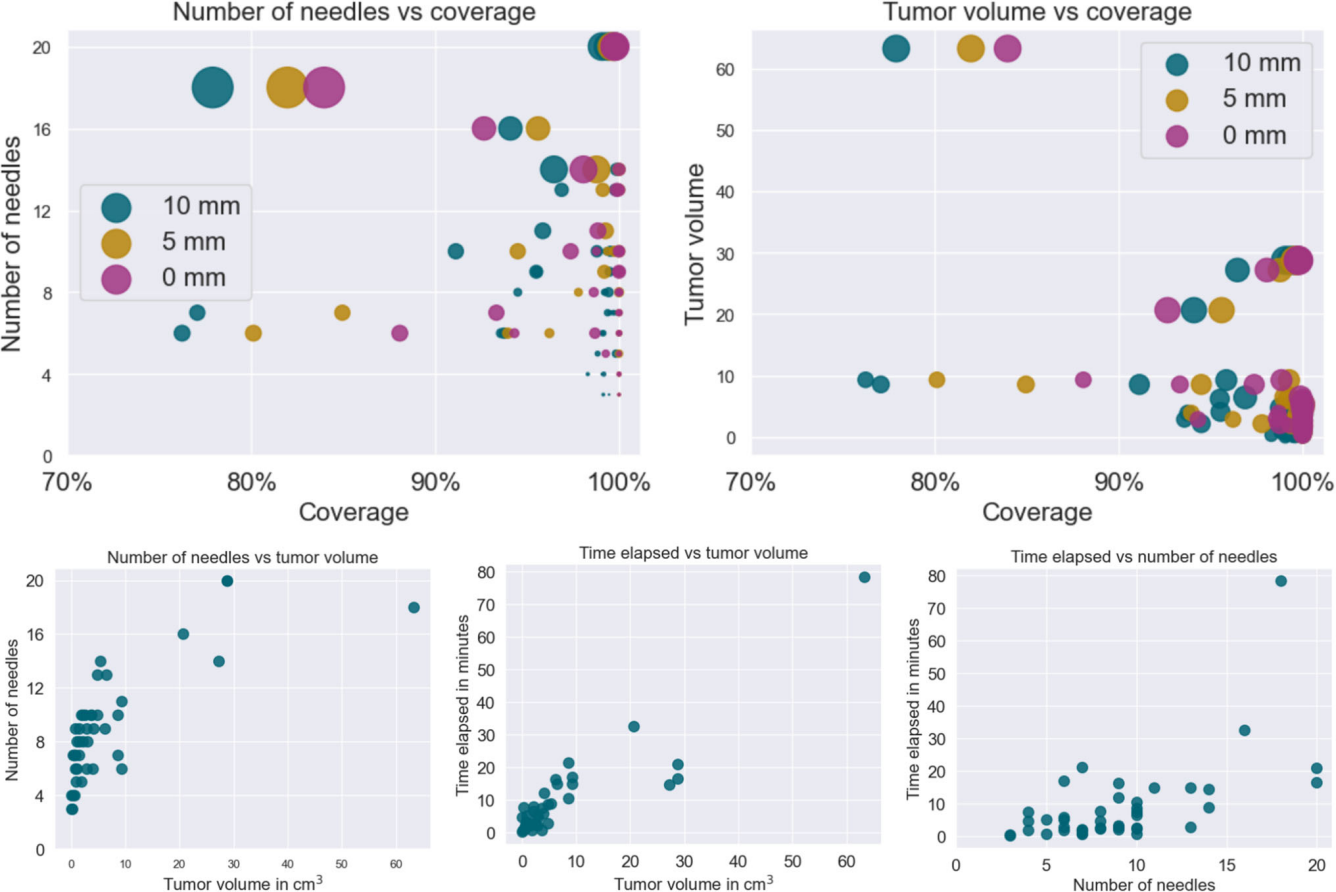
We show scatter plots for visual analysis of different relationships between the variables for solutions in the in-house dataset. The label box in the top row plots shows the color code for different tumor margins. The top row left depicts the number of needles versus tumor coverage, where the area of the scatter point corresponds to the tumor volume. The top row on the right shows tumor volume versus tumor coverage, and the area of the scatter point corresponds to the number of needles. In the bottom row is the number of needles versus tumor volume, time elapsed versus tumor volume, and time elapsed versus the number of needles

## Conclusion & future work

We suggested a rapid and robust method that delivers valid safe solutions to the ablation planning issue, adhering to clinical standards. Our technique can calculate solutions for large tumors utilizing over 10 trajectories simultaneously without any collisions. We rigorously evaluated our approach on 154 liver cancers and clinical cases to demonstrate its efficacy in practical applications.

In the future, we will concentrate on enhancing the algorithm’s performance for large tumors or those situated in difficult locations. We will expand and evaluate our methodology for concurrent planning for multiple tumors. In addition, we will incorporate supplementary vital structures and consider additional clinical constraints. In particular, consider the space required for the insertion guides of the applicators, and the segmentation of intercostal arteries, which are often omitted by automatic segmentation methods due to their small diameter.

## A Supplementary information

### A.1 Pseudocodes

In this section, we provide the pseudocodes for the crossover and mutation operators. Our crossover algorithm is shown in Algorithm 2. Our proposed crossover is a binary crossover that generates two offspring from two parents. If one of the parents is the best individual, we deliberately keep this and create only the second offspring. This ensures that the algorithm focuses on and improves the best solution and supports convergence of the optimization process, but it does not promote the diversity of the population. The *crossoverUniform* in Algorithm 2 distributes the needles evenly in the treatment volume always keeping the minimal distance between needles larger than ablation radius, achieving the best coverage with the minimal number of needles. On the contrary, the *crossoverRandom* picks a random subset of trajectories from the parents for the child. This diversifies the population and keeps low computation time.

**Algorithm 2:**
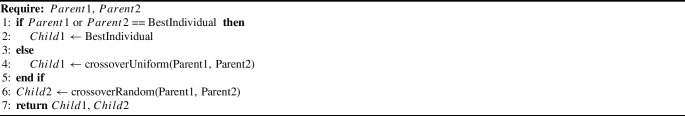
Crossover

**Algorithm 3:**
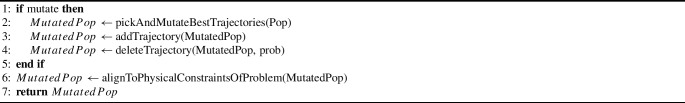
Mutation

Our mutation presented in Algorithm 3 consists of multiple steps. The method selectively identifies trajectories that collectively optimize coverage, while the insertion and target sites of each needle are individually adjusted to increase distance from essential structures or to enhance coverage of the treatment volume.

Then, with a certain probability, the worst-performing needle in the solution is removed, or an additional needle is added if the treatment volume coverage is insufficient. Ultimately, the solution is calibrated to the abdominal environment, ensuring that the trajectories are neither excessively long (> 150 mm) nor excessively short (< 5 mm in the liver), and that each entry point is positioned on the skin while each target is located within the tumor treatment volume.

### A.2 Additional discussion of the clinical dataset

We present the 3D renderings of the Case 1 discussed in Results section in Table 1. The automatic solutions for Case 1 consisted of 7, 6, and 5 trajectories achieving over $96.5\%$ coverage of the tumor and 5 mm safety margin, whereas the clinical solution consisted of 5 trajectories and achieved $94.8\%$ coverage. The comparison of the trajectories in 3D can be seen in Fig. 7. For clarity, we omit critical structures such as ribs or vasculature in the rendering.

Case 2 presents a substantial tumor situated subcapsularly next to the diaphragm and the lungs. Furthermore, the tumor is next to a large hepatic artery. Consequently, it is a rather complex case. Figure 8 provides an optical representation of the tumor’s position relative to the liver and arteries. The tumor coverage and its 5mm safety margin were 75.5% for the clinical approach and 80.1% for the automatic solution in this instance. The inadequate coverage in the clinical solution likely results from segmentation mistakes or inaccuracies in manual needle placements; nonetheless, the actual coverage throughout the process was adequate.

Figure 9 depicts the correlations among variables over various trajectories, coverage, and computation time. The duration for the large tumor over 60 cm$^3$ was 80 minutes. The graphs in the second row demonstrate a positive correlation , consistent with the expectation that larger tumors require more trajectories, hence necessitating additional planning time for the algorithm. Nonetheless, the duration required for computation is also dependent upon additional parameters, including the tumor’s location and its proximity to adjacent vital structures.

## Data Availability

The publicly available dataset 3D-IRCADb-01 [1] was used. Due to legal and privacy restrictions, the in-house dataset used in this study is not publicly available.

## Code Availability

Code is available at https://git.i-med.ac.at/hno/srfa_trajectory_planning_with_ga/.

## Declarations

### Copyright and Licensing

For open access purposes, the author has applied a CC BY public copyright license to any author accepted manuscript version arising from this submission.
